# Application of Deep Learning Algorithms to Visual Communication Courses

**DOI:** 10.3389/fpsyg.2021.713723

**Published:** 2021-09-29

**Authors:** Zewen Wang, Jiayi Li, Jieting Wu, Hui Xu

**Affiliations:** ^1^Pan Tianshou College of Architecture, Art and Design, Ningbo University, Ningbo, China; ^2^Department of Control and Computer Engineering, Polytechnic University of Turin, Turin, Italy; ^3^Engineering University of Armed Police Force, Urumqi, China; ^4^College of Education, University of Perpetual Help System DALTA, Manila, Philippines

**Keywords:** deep learning, visual communication courses, fast style transfer network, image style transfer, TensorFlow

## Abstract

There are rare studies on the combination of visual communication courses and image style transfer. Nevertheless, such a combination can make students understand the difference in perception brought by image styles more vividly. Therefore, a collaborative application is reported here combining visual communication courses and image style transfer. First, the visual communication courses are sorted out to obtain the relationship between them and image style transfer. Then, a style transfer method based on deep learning is designed, and a fast transfer network is introduced. Moreover, the image rendering is accelerated by separating training and execution. Besides, a fast style conversion network is constructed based on TensorFlow, and a style model is obtained after training. Finally, six types of images are selected from the Google Gallery for the conversion of image style, including landscape images, architectural images, character images, animal images, cartoon images, and hand-painted images. The style transfer method achieves excellent effects on the whole image besides the part hard to be rendered. Furthermore, the increase in iterations of the image style transfer network alleviates lack of image content and image style. The image style transfer method reported here can quickly transmit image style in less than 1 s and realize real-time image style transmission. Besides, this method effectively improves the stylization effect and image quality during the image style conversion. The proposed style transfer system can increase students’ understanding of different artistic styles in visual communication courses, thereby improving the learning efficiency of students.

## Introduction

Of recent years, with the rapid economic and social developments in China, the public has a new understanding of talent training, and the concept of talent training has also shifted (Wang et al., 2015). The core of talent training in modern society has changed from having students master knowledge to allowing students to adapt to a lifelong learning society; with positive attitudes to master knowledge and skills, students should have the ability to knowledge conversion, critical thinking, and solve practical problems (Craik and Wyatt-Rollason, 2002; Fryer and Vermunt, 2018). These are consistent with the learning methods advocated by deep learning and the overall development theory of people. Although the importance of deep learning and overall development has been emphasized by higher education, the application of deep learning in the actual education process is too weak, which cannot enable students to develop comprehensively (Cannatella and Cannatella, 2018). Many people think that visual communication design is “graphic design,” which is inaccurate (Jun, 2018; Yanuarsari and Setiawan, 2018). Indeed, visual communication design originated from “graphic design” or “printing art design.” With the gradual scope expansion of modern design, however, digital technology has penetrated various fields of visual communication design. Meanwhile, the influence and participation of multimedia technology on art and design keep increasing, and the educational methods of visual communication design have attracted full attention (Kim and Lee, 2016; Delhey and Peters, 2017).

Visual communication design is a form of non-verbal communication. It applies linguistics or semiotics to the teaching of visual communication design, making graphic design an innovative and scientific discipline (Russmann and Svensson, 2017). The utilization of narrative techniques in visual communication design not only stimulates the creativity of designers but also evokes the visual memory of the audiences, thereby promoting bilateral communication between the two entities (Lee et al., 2018). Yang and Hsu (2017) adopted a paired group design, in which 30 participants were divided into an experimental group and a control group and participated in different activities within 4 weeks; their results suggested that incorporating narrative theory into graphic design courses could improve students’ poster designing capabilities, such as theme concepts, image creation, and visual esthetics (Yang and Hsu, 2017). For the current phenomenon of “theoretical curriculum marginalization” of students majoring in arts, Huang et al. (2019) explored the Canadian BOPPPS teaching model, integrated it into their courses, analyzed the problems that occurred during teaching, and finally, optimized the teaching model and method. Visual communication design conveys information to the audiences through visual symbols and visual influences. However, due to the rapid development of digital communication and multimedia technology, the practice of visual communication design encounters broad development prospects and challenges. For an efficient and safe information literacy education model, the hierarchical model of information literacy education is designed through visual communication. The visual image is analyzed based on the data and information of each part of the visual communication in the 3D environment, thereby constructing an information literacy education model (Liu et al., 2016). Chu and Wu (2018) researched the application of in-depth correlation features in image style classification comprehensively, designed various correlations and transformed them into style vectors, explored the classification performance brought by different variants, and showed the effectiveness of in-depth association features; finally, a learning framework to automatically learn the associations between feature maps was proposed.

The above results suggest that research on visual communication courses and image style transfer are various; however, the combination of the two is rarely reported. Hence, the combination of visual communication courses and image style transfer is investigated based on deep learning algorithms. Visual communication design is closely connected to the way of thinking and design philosophy. In some respects, the three are interrelated. Therefore, innovative works on visual communication thinking mode and design concepts should be valued to improve the design quality effectively. Firstly, the main contents of the visual communication course are sorted out, and the relationship between the course and the image style transfer is discussed. Then, a style transfer method is designed based on deep learning. In addition, a fast style transfer network is proposed based on TensorFlow to separate training and execution, which improves the speed of image transfer. Moreover, the image style transfer model is trained.

## Materials and Methods

### Thinking Model of Visual Communication Design

**FIGURE 1:**
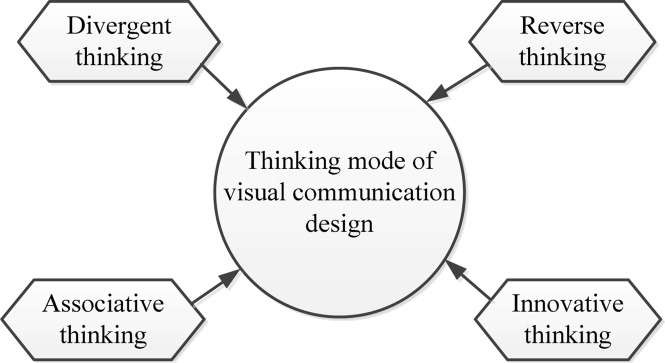
Structure of the thinking model of visual communication design.

The goal of visual communication design is to enhance people’s visual experience and enjoyment. Therefore, in the process of designing, everything should be visual-centric. All design contents should take vision as the starting point and the ending point of further research. Figure 1 illustrates the four aspects of the thinking model of visual communication design.

The visual communication design of divergent thinking is a unique way of thinking for designers while designing. The presentation of the final design is affected by many factors. The designers need to consider whether their works meet the requirements from multiple angles and aspects, as well as whether the works take vision as the center, diverge the thinking, and use the imagination, thereby presenting the infectious and meaningful works. In the process of divergent thinking, designers should be reasonable to ensure that the works are connected to the topic (Forthmann et al., 2020).

Designers must adopt reverse thinking during visual communication design. While designing, it is necessary to enrich the connotation of the works from all aspects to improve the innovation and final effect of the works. Therefore, designers need to reasonably incorporate excellent design cases in daily life into their design works. Besides, designers can integrate available materials through reverse thinking for the inspiration of work designs.

The associative thinking of visual communication design is widespread. It refers to linking other things with particular things during designing and generating other creative inspirations during associative thinking, which can effectively improve the connotation and innovation of works (Boot et al., 2017; Krause et al., 2017). The connection between things in associative thinking can be a causal connection, close connection, or contrast connection. In practical applications, designers can choose particular connections according to their needs, thereby improving the visual communication effects of the works.

The innovative thinking of visual communication design refers to that when people appreciate the design works, they will understand the works through information such as color, shape, and text. Therefore, the innovative thinking of artistic language can well indicate the thinking model of innovative vision.

### Image Style Transfer Based on Deep Learning

During the teaching process of visual communication courses, the rendering of artistic style is particularly important. Different rendering methods will present different effects and different visual perceptions for the audiences (Yang and Hsu, 2017; Saterbak et al., 2018). Therefore, the image style transfer network is designed through deep learning methods to provide better visual communication courses. Recently, artificial intelligence technology led by deep learning has begun to be applied more widely in various fields of society. The cross-collision of artificial intelligence and art has attracted considerable attention in technical fields and artistic fields. Various image processing software and filter applications developed based on the above technologies have attracted numerous users once they were launched (Shrivakshan and Chandrasekar, 2012; Dutta et al., 2013; Desai et al., 2020). The core of all kinds of wonders is the image style transfer based on deep learning.

The image style transfer is the process of obtaining an image with a converted style with a given original image and style image. The original image indicates the content, while the style image indicates the style; the obtained image with the converted style indicates the generated image (Sun et al., 2017).

The fast style transfer network consists of two parts. One is the image transform network, and the other is the loss network.

The input layer in the image transform network receives an input image, and the output of the final output layer is also an image (the result of style transfer). The overall model is divided into two stages, namely the training stage and the execution stage.

In the training stage, a style image is chosen. During the training process, the images in the dataset are input to the network. Then, the image transform network generates the resulting image y, the loss network extracts the feature map of the image, and the generated image y is separately calculated with the target style image y_s_ and the target input image (content image) y_c_ for loss calculation. Finally, the weight of the image transform network is adjusted according to the loss value, and the target effect is achieved by minimizing the loss value.

In the execution stage, an image is given, which is input into the trained image transform network, and the result of the image style transfer is output. The image transform network is essentially a Convolutional Neural Network (CNN) (Liu et al., 2018; Yang et al., 2019; Wu et al., 2021). Here, the image transform network is a deep residual network without any pooling layer. Instead, it uses stride convolution or micro stride convolution for up-sampling or down-sampling. The neural network here consists of five residual blocks. Except for the last output layer, all non-residual convolution layers are followed by a spatial instance-normalization and the non-linear layer of ReLU. The instance-normalization regularization prevents overfitting.

The last layer uses a scaled Tanh to ensure that the pixels of the output image are between [0, 255]. Except that the first and last layers use a 9 × 9 convolution kernel, all other convolution layers use a 3 × 3 convolution kernel.

The Loss Network φ can define content loss and style loss to measure the gap between the content and the style, respectively. Each input image x has a content target y_c_ and a style target y_s_. For style transfer, the content target yc is the input image x and the output image y. The style y_s_ should be combined with the content x = y_c_. The system trains a network for each target style.

It is essential for image classification to train the CNN in advance to clarify the shortcomings of the pixel-by-pixel loss function and ensure that the utilized loss function can better measure the gap in image perception and semantics (Wang et al., 2018). This CNN has learned to perceive and encode semantic information, which is precisely what needs to be done in the loss function of the image style transfer system. Hence, a pre-trained network φ for image classification is utilized to define the loss function of the system. Then, the same loss function of the deep convolutional network is utilized for training the proposed deep convolutional transfer network.

Although the loss network here is also a CNN, the parameters are not updated. It is only utilized to calculate content loss and style loss. The training update is the weight parameters of the previous image transform network. Therefore, from the perspective of the entire network structure, the input image is transferred through the image transform network. Then, the corresponding loss is calculated. The entire network continuously updates the weights of the previous image transform network by minimizing this loss.

For the process of finding the loss, instead of constructing the loss function by pixel-by-pixel difference, the perceptual loss function is applied to extract advanced features from the pre-trained Loss Network. During the training process, the perceptual loss function is more suitable for measuring the similarity between images than the pixel-by-pixel loss function.

(1) Content loss: two perceptual loss functions are designed to measure the advanced perceptual and semantic difference between two images. The content loss calculation employs a VGG calculation to represent the advanced features (content). Because the VGG model is initially used for image classification, a trained VGG model can effectively extract the advanced features (content) of the image (Ha et al., 2018; Tammina, 2019; Hameed et al., 2020).

The initial image is represented as _$\vec{P}$_, and the new image after processing is represented as _$\vec{X}$_. Let *F*_1_ and *P*_1_ be the feature representation in the *l* layer, respectively. The residual sum of squares loss function between the two can be expressed as Eq. 1.


(1) $$L_{content}\left(\vec{p},\vec{x},\vec{l}\right)=\frac{1}{2}\sum_{i,j}{\left(F_{i,j}^{i}-P_{i,j}^{l}\right)}^{2}$$

This function indicates that for the image *p* for content extraction, the content represented by the position is denoted as *P*, and through constructing _$\vec{x}$_, the characteristics of the corresponding position are infinitely close to *P*, to finally achieve the minimum content loss function. Its reciprocal can be written as Eq. 2.


(2) $$\frac{\partial L_{content}}{\partial F_{i,j}^{i}}=\left\{ \begin{array}{c} \left(F^{l}-P^{l}\right),ifF_{i,j}^{i}>0\\ 0,ifF_{i,j}^{i}<0 \end{array}\right.$$

The image is modified until the same feedback is obtained as the initial image at a CNN layer.

Gram matrix _*G*_1_ ∈ *R*^*N*_1_×*N*_*l*_^_ is used to represent the feature relationship. The relationship between different network levels is different, and the texture information except the overall structure can be obtained. The point product _$G_{i,j}^{i}$_ of *i* and *j* the layer in the *l* layer of the feature map can be written as Eq. 3.


(3) $$G_{i,j}^{i}=\sum_{k}F_{i,k}^{1}F_{i,k}^{l}$$

The selected image type is used to create new images, to obtain the information of feature space constructed in the CNNs at different levels. The original image is represented as _$\vec{a}$_, the new image is represented as _$\vec{x}$_, and the style of one layer is represented as *A*^1^. *G*^1^ denotes the ratio of the *l* layer to the overall loss. *E*_1_ and the total style loss function *L*_*style*_ are, respectively, expressed as:


(4) $$E_{1}=\frac{1}{4N_{l}^{2}M_{l}^{2}}\sum_{i,j}{\left(G_{i,j}^{i}-G_{i,j}^{l}\right)}^{2}$$

(5) $$L_{style}\left(\vec{a},\vec{x}\right)=\sum_{l=0}^{L}w_{l}E_{l}$$

In the migration of image style, a new image can be obtained by combining the content representation in the image _$\vec{p}$_ and the style representation in the image _$\vec{a}$_. This method can represent the style image _$\vec{a}$_ in the content image _$\vec{p}$_. The minimization function *L*_*total*_ can be presented as:


(6) $$L_{total}\left(\vec{p},\vec{a},\vec{x}\right)=\alpha L_{content}\left(\vec{p},\vec{x}\right)+\beta L_{content}\left(\vec{a},\vec{x}\right)$$

where α and β refer to the proportion of content and style of the image.

Through the future extraction content image and style image by CNN and the visualization of the convolution layer of the network, the activation state of each layer of CNN corresponds to specific information. Different filters activate different contents, showing various activation values, and finally visualize them as different images.

### Specific Implementation of the Fast Style Transfer Network

TensorFlow is the second-generation artificial intelligence learning system developed by Google based on DistBelief (Lozano Jimenez et al., 2019; Sun et al., 2021). Its name comes from its operating principle. Tensor means an N-dimensional array, while Flow means the calculation based on the data flow graph. Therefore, TensorFlow is the calculation process of tensor flowing from one end of the flow graph to the other end. TensorFlow is a system that transmits complex data structures to artificial intelligence neural networks for analysis and processing.

TensorFlow can be employed in various fields of deep machine learning, such as speech recognition and image recognition (Lemley et al., 2017). The deep learning infrastructure DistBelief developed in 2011 has been improved in various aspects. It can run on small devices, such as smartphones, and large devices, such as data center servers with thousands of equipment. TensorFlow is completely open-source; anyone can use it.

The image transform network contains three convolution layers at the beginning; these layers are implemented by the custom function conv layer, in which the tf.nn.conv2d function provided by TensorFlow is called for convolution operation. Then, the five residual modules are implemented by the custom function residual block. The input image size should be consistent with the output. Correspondingly, the next three deconvolution operations are implemented by the custom function _conv_tranpose_layer, which invokes the tf.nn.conv2d_transpose function in the framework. A Tanh activation function is set at the end of image transform network to map the output value to (0, 255).

### Simulation Experiments

Images of different styles are downloaded from Google Gallery to train the model. After training different style models, the time of model training can be saved, and the real-time image style rendering can be performed directly. The six types of images selected from Google Gallery are landscape images, architecture images, character images, animal images, cartoon images, and hand-painted images. The styles of different types of images are transferred, and the length and loss function value of the style transfer are recorded. In the network training process, the batch size is 8, the number of epochs is 8, the learning rate is 0.0001, and the Adam optimization algorithm is used for parameter back propagation.

In the style transfer network, the encoder is mainly responsible for extracting the features of the image under different convolution layers to prepare for the subsequent multi-scale feature fusion. The encoder network uses the pre-trained VGG network structure from conv1_1 to relu4_1. The convolution kernel size is 3 × 3. Each convolution layer is followed by a relu activation function, and there is a maximum pooling layer after relu1_2, relu2_2 and relu3_4 to perform down sampling for the feature map. Considering the small gap between the original image and the image after only one layer of convolution, the weighted image may restrict the embedding of style and affect the quality of style transfer, so the relu1_1 result is not fused when weighted.

**FIGURE 2:**
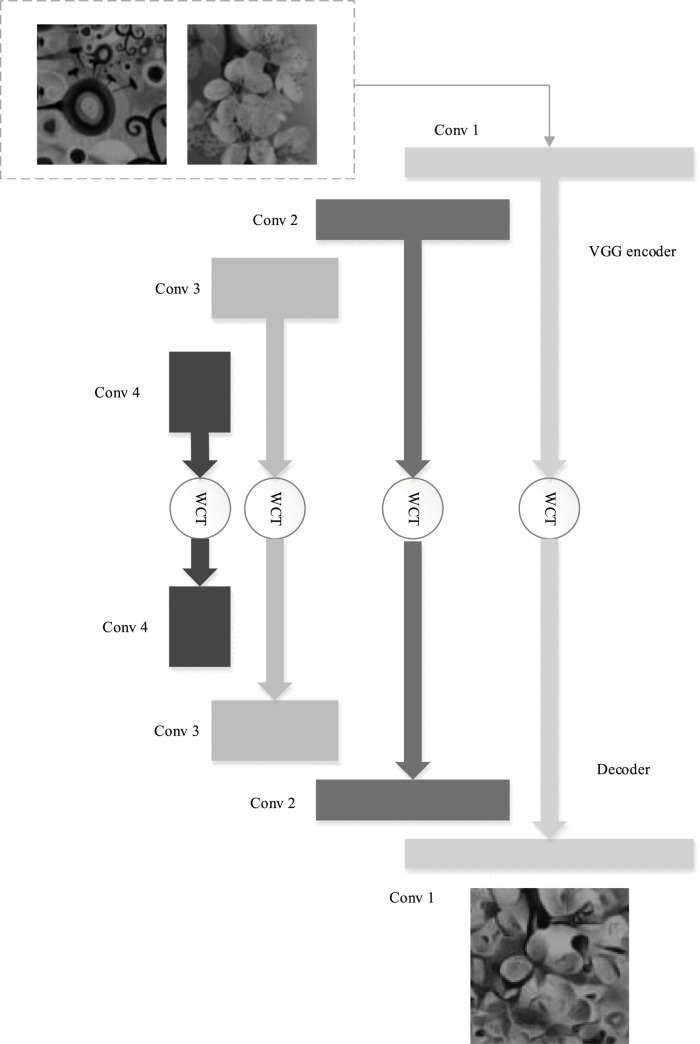
Structure of the multi-scale feature fusion network.

The whole network style transfer process is as follows. Firstly, the content image and the target style image are sent into the pre-trained VGG encoder to extract the multi-scale features at different levels. Then, the content feature map and style feature map of the encoder at relu2_1, relu3_1 and relu4_1 are transformed by whitening and coloring, respectively, to obtain the fusion feature maps with different sizes and scales. Finally, the fusion feature maps of different scales are weighted and fused in the decoder, which is mapped back to the original pixel RGB space to obtain the style transfer result. Figure 2 illustrates the structure of the multi-scale feature fusion network.

## Results and Discussion

### Results of Image Style Transfer

**FIGURE 3:**
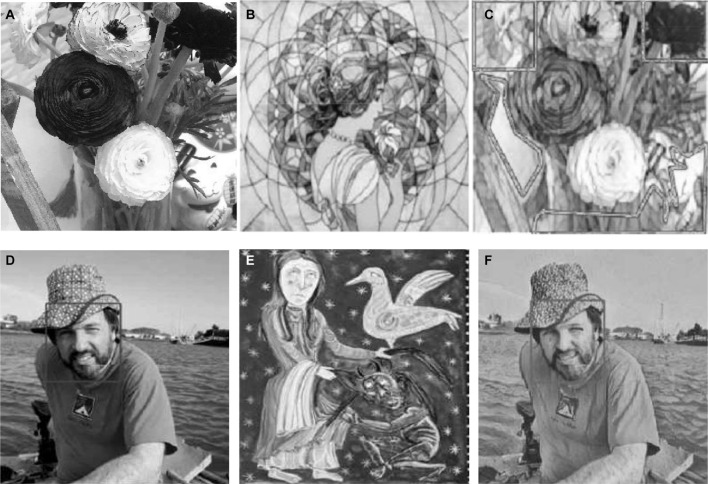
Results of image style transfer. **(A,D)**: content image; **(B,E)**: style image; **(C,F)**: results of image style transfer.

Figure 3 reveals the results of image style transfer.

The marked area in Figures 3C,F is a complex area for style transfer. In the designed image style transfer network, the image style can be better transferred, and the style of the image is consistent with the style image. Figure 3 illustrates that the image has changed in style, which is no longer the style of the original content image; instead, the style is transformed into a style that is close to the style image in terms of utterance. The style of the original content image belongs to realism. After many iterations, the image output by the image transform network is similar to the style image, which is more abstract and cubist.

### Test Results of the Model Loss Function

Figure 3 illustrates the test results of the content loss function of the model with different times of iterations.

**FIGURE 4:**
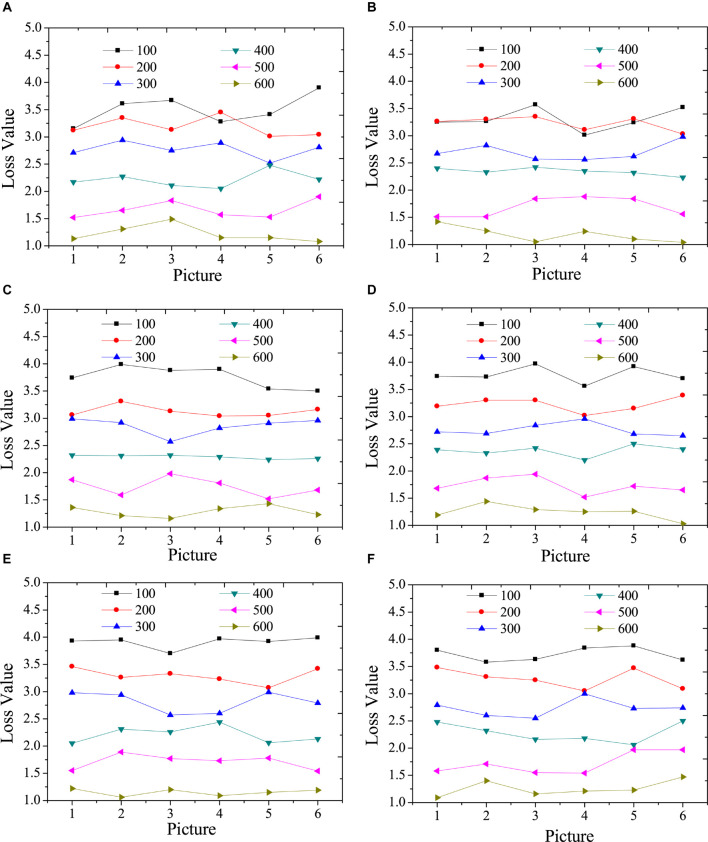
Test results of content loss function under different iterations. **(A)**: landscape images; **(B)**: architecture images; **(C)**: character images; **(D)**: animal images; **(E)**: cartoon images; **(F)**: hand-painted images.

Figure 4 indicates that the higher the iteration numbers are, the greater the overall loss function is reduced. However, the reduction trend of different categories is not the same; thus, in the actual application of the model, the number of categories can be selected artificially. A model with a small loss function and an appropriate number of iterations can be accurately identified and can reduce the calculation time. In addition, in terms of the content loss of the model, the larger the iteration numbers are, the smaller the content loss is. However, the increase in the number of iterations represents the increase in the style transfer time of a single image. Hence, the iteration numbers of the model should be determined according to the complexity of the specific image in practical applications.

Figure 4 provides the test results of the style loss function of the model under different iterations.

**FIGURE 5:**
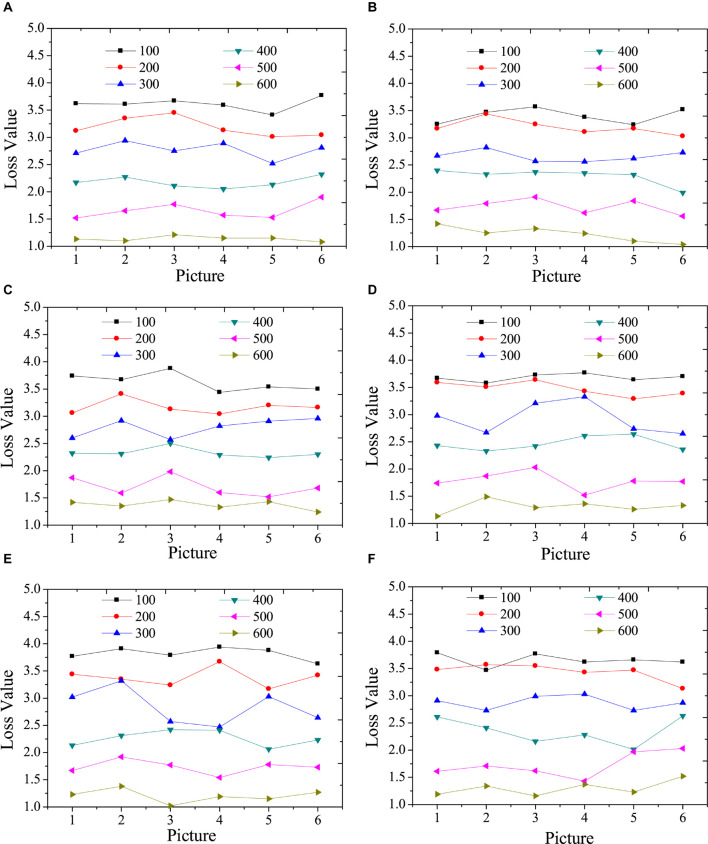
Test results of style loss function under different iterations. **(A)**: landscape images; **(B)**: architecture images; **(C)**: character images; **(D)**: animal images; **(E)**: cartoon images; **(F)**: hand-painted images.

Figure 5 suggests that the higher the iteration numbers are, the greater the overall loss function decreases. In addition, in terms of the style loss of the model, the larger the iteration numbers are, the smaller the style loss is. However, the increase in the number of iterations represents the increase in the style transfer time of a single image. Hence, the iteration numbers of the model should be determined according to the complexity of the specific image in practical applications.

### Results of Time Consumption of Image Style Transfer

Figure 5 displays the time consumption of the image style transfer for a single image.

**FIGURE 6:**
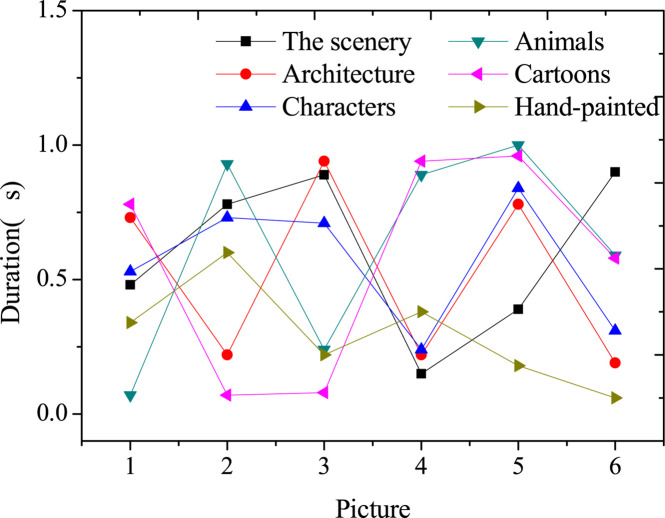
Time consumption of image style transfer.

Figure 6 reveals that the proposed method of image style transfer takes less than 1 s, which can quickly transfer the image style and achieve the real-time image style transfer. Besides, the transfer effect is good, and the expected goal is achieved.

## Discussion

Visual communication design conveys information to the audiences through visual media performance. It is “a design for people to see and an information design.” Visual communication is generally summarized as four procedures: “who,” “what,” “to whom,” and “effects and impacts.” In the daily lives of people, many fields are involved in visual communication design, such as television, film, architecture, plastic arts, various design products, and various icon, stage, and text designs. Since images of different styles will bring different experiences to audiences, students enrolled in visual communication courses need to understand the utterance of images in various styles accurately. Recently, artificial intelligence technologies led by deep learning have been applied more widely in various fields of society. Among them, the cross-collision of artificial intelligence and art has attracted considerable attention in many research fields. Image style transfer based on deep learning has become one of the current research hot spots.

Therefore, a real-time style transfer system is designed to facilitate the implementation of the visual communication courses by teachers. The experimental results reveal that for the difficult-to-transfer parts, the designed image style transfer system can transfer the image styles well, illustrating the effectiveness of the system. Besides, as the iteration numbers increase, the lack of image content and image style can be improved, showing that for images with different difficulties in style transfer, different iteration numbers can be chosen for efficient image style transfer. Moreover, the designed image style transfer system consumes a shorter time in transferring various types of images, thereby achieving real-time transfer. Cai et al. (2019) proposed a transfer method of image color style based on color feature extraction for mobile applications; this method used dichotomy to extract the color features of the template image; then, it employed the Quartz2D engine of iOS based on the color features of the template image to draw the target image; finally, it utilized the iOS Metal interface to adjust the color saturation of the target image and sharpen the edge of the target image; during the entire process, the color style of the source image was transferred to the new image, realizing the transfer system of image color style for IOS applications (Cai et al., 2019). However, it only transfers the image color. In constant, the effect of the proposed style transfer system is more excellent.

The following suggestions are put forward for the setting of visual communication courses. First, in China, it is a common phenomenon in higher education institutions that schools emphasize theories rather than practices. The low usability of courses makes students unable to apply the knowledge they learned to practical works. Therefore, for course settings, Chinese colleges and universities should integrate theoretical knowledge with market demands and student needs, and take student development as the principal teaching goal. For the content of the courses, practices should be the leading factor to cultivate students’ innovative thinking and practical skills, as well as guiding students to study instead of blindly instilling theoretical knowledge into students and not paying attention to students’ practical application abilities.

## Conclusion

The multi-scale feature style transfer algorithm based on deep learning is reported here, and the feasibility of this method is proved through the style transfer effect realized by different network parameters. The designed rendering system can assist the teaching of visual communication courses by teachers. Although some fruitful results are achieved in this experiment, some shortcomings exist in the experimental process. On the one hand, the designed system can only perform style rendering of a limited model that has been trained, but it cannot support arbitrary style rendering. On the other hand, the model training consumes a considerable amount of time, which cannot feedback random style rendering results to users. Therefore, in future, the method of training style models will be explored to reduce the time required for model training.

## Data Availability Statement

The raw data supporting the conclusions of this article will be made available by the authors, without undue reservation.

## Ethics Statement

The studies involving human participants were reviewed and approved by Ningbo University Ethics Committee. The patients/participants provided their written informed consent to participate in this study. Written informed consent was obtained from the individual(s) for the publication of any potentially identifiable images or data included in this article.

## Author Contributions

All authors listed have made a substantial, direct and intellectual contribution to the work, and approved it for publication.

## Conflict of Interest

The authors declare that the research was conducted in the absence of any commercial or financial relationships that could be construed as a potential conflict of interest.

## Publisher’s Note

All claims expressed in this article are solely those of the authors and do not necessarily represent those of their affiliated organizations, or those of the publisher, the editors and the reviewers. Any product that may be evaluated in this article, or claim that may be made by its manufacturer, is not guaranteed or endorsed by the publisher.
